# Current Perspectives on Periodontitis in Systemic Sclerosis: Associative Relationships, Pathogenic Links, and Best Practices

**DOI:** 10.3390/diagnostics13050841

**Published:** 2023-02-22

**Authors:** Andreea Ciurea, Nicolae Voicu Rednic, Andrada Soancă, Iulia Cristina Micu, Alina Stanomir, Diana Oneț, Petra Șurlin, Ileana Filipescu, Alexandra Roman, Ștefan Ioan Stratul, Cristina Pamfil

**Affiliations:** 1 Department of Periodontology, Faculty of Dental Medicine, Iuliu Hațieganu University of Medicine and Pharmacy Cluj-Napoca, Victor Babeș St., No. 15, 400012 Cluj-Napoca, Romania; 2 Department of Gastroenterology, Iuliu Hațieganu University of Medicine and Pharmacy Cluj-Napoca, “Prof. Dr. Octavian Fodor” Regional Institute of Gastroenterology and Hepatology, Croitorilor St., No. 19, 400394 Cluj-Napoca, Romania; 3 Department of Periodontology, University of Medicine and Pharmacy Craiova, Petru Rareș St., No. 2, 200349 Craiova, Romania; 4 Department of Rheumatology, Iuliu Hațieganu University of Medicine and Pharmacy Cluj-Napoca, Clinicilor St., No. 2, 400000 Cluj-Napoca, Romania; 5 Department of Periodontology, Faculty of Dental Medicine, Anton Sculean Research Center for Periodontal and Peri-Implant Diseases, Victor Babeș University of Medicine and Pharmacy Timișoara, Revoluției from 1989 St., No. 9, 300041 Timișoara, Romania

**Keywords:** periodontitis, systemic sclerosis, pathogenesis, dental biofilm

## Abstract

Systemic sclerosis is a chronic, autoimmune, multisystemic disease characterized by aberrant extracellular matrix protein deposition and extreme progressive microvasculopathy. These processes lead to damage within the skin, lungs, or gastrointestinal tract, but also to facial changes with physiognomic and functional alterations, and dental and periodontal lesions. Orofacial manifestations are common in SSc but are frequently overshadowed by systemic complications. In clinical practice, oral manifestations of SSc are suboptimally addressed, while their management is not included in the general treatment recommendations. Periodontitis is associated with autoimmune-mediated systemic diseases, including systemic sclerosis. In periodontitis, the microbial subgingival biofilm induces host-mediated inflammation with subsequent tissue damage, periodontal attachment, and bone loss. When these diseases coexist, patients experience additive damage, increasing malnutrition, and morbidity. The present review discusses the links between SSc and periodontitis, and provides a clinical guide for preventive and therapeutical approaches in the management of these patients.

## 1. Introduction

Systemic sclerosis (SSc), or scleroderma, is a chronic, autoimmune, multisystemic disease characterized by aberrant extracellular matrix protein deposition and extreme progressive microvasculopathy [1]. These processes lead to severe organ damage within the skin, lungs, or gastrointestinal tract, but also to facial changes with physiognomic, and functional alterations and dental and periodontal lesions [2,3,4].

Orofacial manifestations are common in SSc (up to 80%) but are frequently overshadowed by systemic complications [2]. In SSc, the oral involvement initiates and maintains a vicious circle leading to malnutrition: fibrosis with secondary xerostomia favors tooth decay and periodontitis, leading to impaired mastication, dysphagia, and poor nutritional status. These features, in addition to gastroesophageal reflux and malabsorption, contribute to significant morbidity and gastrointestinal-associated mortality in SSc [5].

The limitation in mouth opening induced by fibrosis is associated with difficulties in performing examinations (manometry, fibroscopy) or intubations, which hampers the assessment and treatment of SSc patients [6].

In clinical practice, oral manifestations of SSc are infrequently addressed, and their management is not included in the general treatment recommendations [7,8]. Increased awareness of oral manifestations, including periodontitis, and further research, are essential to improving patient outcomes.

Periodontitis is a chronic, complex, multi-factorial infectious disease in which subgingival dysbiotic biofilm triggers a local, usually excessive, non-resolved inflammation that leads to the destruction of periodontal tooth-surrounding tissues. Periodontitis is a highly prevalent disease affecting around 50% of the population [9,10,11,12,13], with its severe forms affecting globally 11% of the population [14]. The continuous loss of periodontal attachment and alveolar bone results in some clinical signs such as gingival recessions, increasing clinical crowns, secondary tooth migrations, tooth mobility, furcation lesions, and finally to tooth loss and severe oral dysfunction.

Periodontitis has been associated with several systemic conditions, such as diabetes mellitus, cardiovascular disease, or pregnancy-related adverse effects. Other associations have been reported with neurodegenerative and autoimmune diseases [2,15,16,17,18,19].

Evidence from epidemiological studies suggests that periodontitis is more common in patients with inflammatory rheumatic diseases such as rheumatoid arthritis [20,21,22]. Patients with rheumatoid diseases and periodontitis share common pathogenic characteristics mostly related to an imbalanced chronic inflammation and autoimmunity responsible for tissue degradation and loss of function [23,24,25,26]. The causal relationship between the two entities is supported by interventional studies, which indicate that periodontal therapy reduces rheumatic disease activity and severity [21,27].

A reciprocal cause-and-effect relationship between periodontitis and SSc has been described. Periodontitis in SSc could aggravate general disease-induced disability through tooth mobility or loss of teeth, masticatory and aesthetic dysfunctions, and profoundly impairs patients’ quality of life. On the other hand, dysfunctionalities in SSc prevent optimal dental hygiene, which aggravates periodontal destruction [28]. Moreover, the treatment of periodontitis may be impossible to perform in the late stages of SSc due to severe limitation of mouth opening; thus, the utmost importance of periodontal screening, prophylaxis, and treatment in early SSc stages.

Although some pathogenic links between rheumatic diseases and periodontitis are specified in the literature, the pathogenic intersections between SSc and periodontitis are less obvious and less organized [24,26], requiring a systematization of data on this topic. In addition, the therapeutic approaches of periodontitis in patients with SSc are relatively vaguely specified and there is no published exhaustive therapeutic plan that logically addresses the entire oral pathology of these patients [28,29,30]. Patients with SSc and periodontitis need individualized, gradual therapy based on the latest recommendations of the literature [31,32].

The present review aims to provide evidence-based data on the SSc-periodontitis associations, to review data supporting the biological plausibility of the links between SSc and periodontitis, and to provide a clinical guide including preventive and therapeutical approaches for the management of periodontitis in SSc patients.

## 2. Oral Manifestations in Systemic Sclerosis

Orofacial involvement in SSc is associated with significant morbidity and reduced quality of life [33]. The fibrosis of the salivary glands determines reduced salivary flow, negatively impacting mastication and nutrition. Xerostomia aggravates dysphagia and impairs natural oral self-cleaning, thus raising the incidence of tooth decay, erosions, periodontitis [34], and *Candida albicans* infections [35].

Tongue rigidity is among the first acknowledged clinical manifestations in SSc and hinders speaking and swallowing [36]; facial fibrosis leads to a classic mask-like face, with loss of wrinkles, thin white lips, and microstomia [36].

Telangiectasia of facial skin and oral mucous membranes is the result of an aberrant attempt at increasing blood perfusion to hypoxic tissues secondary to capillary loss and failed vascular repair [37].

Mandibular bone resorption is described as resorptive lesions of the mandibular angles, coronoid processes, and zygomatic arches at the sites of muscle attachment [38]. Mandibular bone resorption has been reported in 20–33% of radiographically examined patients [39] and occurs predominantly in diffuse SSc [39]. Incriminated factors include muscle contractures secondary to fibrotic changes, microvasculopathy, and pressure ischemia due to thickened skin and muscle atrophy [40]. Atrophic and fibrotic alterations of the synovia may also induce temporomandibular joint disease [40]. In time, these muscular, osseous, and synovial changes hinder mandibular movements, limit the mouth opening, and decrease the interincisal distance in SSc patients [41].

Microstomia occurs due to sclerosis of perioral soft tissues; it impairs mastication, proper oral hygiene, and social relationships. Importantly, reduced oral aperture rends dental treatments more difficult or sometimes impracticable [41]. Different proposed regimens of oral aperture augmentation and stretching exercise programs were associated with contradictory results related to the improvement in orofacial functions. Several variables related to the type of exercises, duration, number of repetitions, teaching method, and patient compliance could explain the reported inconsistencies [42,43].

The Mouth Handicap in Systemic Sclerosis (MHISS) allows a homogeneous and adequate identification, and evaluation of the limitations in mouth functioning. This short, easy-to-fill-out questionnaire evaluates the degree of mouth disability according to three domains: mouth-opening restriction, mouth dryness, and aesthetic concerns [44]. Studies that employed MHISS as a measure of the quality of life showed that oral features are associated with global disability and negative perception of health status [45].

SSc is associated with a higher incidence of periodontitis. Characteristic periodontitis-related signs identified at oral examination should prompt a specialized assessment [46]. Notably, fibrosis, especially when it involves the frenum, can promote gingival recessions unrelated to periodontitis [36]. Malocclusion has also been described in some SSc patients [47].

## 3. Oral Hygiene in SSc Patients

SSc disease-related factors, such as xerostomia, diminished oral aperture, and manual dexterity impairments, have been reported to impact oral hygiene behavior in these patients. The handling difficulties of interdental cleaning devices were associated with less than daily interdental hygiene. Conversely, evening dental flossing reduced gingival inflammation in SSc [48]. Beyond the factors mentioned above, depressive symptoms common in SSc patients [49] are associated with disinterest in performing oral hygiene, less likelihood to brush teeth at least twice daily, and high values of plaque indices [46]. Neglected oral hygiene should alert dentists of possible underlying depression, and prompt patient referral for evaluation and specialized care [49].

In SSc, adapted oral hygiene devices are recommended to accommodate microstomia and reduced manual dexterity [48]. An electric toothbrush with a small head can facilitate oral hygiene, improve plaque control, and mitigate gingival inflammation [50]. The round, small-sized head of electric brushes (working in an oscillating-rotating action and associated with vibrating movements) is smaller than the head of manual brushes, can facilitate overall access, and individualizes hygiene according to the level of gingival margins. Dentists should recommend at least twice daily tooth brushing to prevent oral diseases.

SSc patients should be educated on the importance of daily flossing [48]. Adapted flosser over finger flossing is preferred in patients with reduced manual dexterity, as it facilitates patient compliance and long-term regular interdental cleaning habits [51]. The choice of an adapted flossing device or interdental brushes could increase easiness to use and daily interdental hygiene adherence [48].

## 4. Association between SSc and Periodontitis Supported by Clinical Observational Studies

Although orofacial manifestations in SSc have long been acknowledged, only a scarce number of observational studies assessed the periodontal status in SSc.

Many case-control studies reported that SSc was associated with impaired periodontal health defined based on pathological parameters of periodontitis (periodontal pockets, clinical attachment loss, percentage of deep pockets, local inflammation, gingival recessions, widening of periodontal space) or on various periodontitis case definitions [20,52,53,54,55,56,57,58,59] valid at the time of reports. In view of the various employed definitions of periodontitis in clinical studies, or the use of surrogate parameters, the epidemiological data supporting the association between SSc and periodontitis is hard to compare or to draw conclusions.

There is a group of clinical studies that have observed the associative relationship between SSc and periodontitis by analyzing only some surrogate parameters related to periodontal destruction. A higher prevalence of periodontal pockets and periodontal attachment loss in SSc than in healthy individuals was reported, indicating a possible relationship between SSc and periodontitis [20]. The increased loss of periodontal attachment in SSc remained significant after adjustment for known risk factors and indicators of periodontitis, such as age, gender, education, smoking status, alcohol consumption, and plaque index. The reduced periodontal bleeding on probing and gingival inflammation in SSc likely reflects the underlying fibrosis and capillary loss in SSc [20]. Conversely, other studies highlighted significantly higher bleeding on probing indices, witnessing more local inflammation in SSc patients than in controls; however, the cohorts included fewer and younger SSc patients and excluded patients with xerostomia, edentulous patients, and smokers. Significantly higher mean full-mouth probing depth dimensions [52,54], and percentage of sites with probing depths of 4 to 5 mm [52] were also reported in SSc patients than in controls.

SSc patients exhibited higher odds of increased probing depth compared with controls, even after adjustment for age, sex, and the presence of major organ involvement, as well as a higher prevalence of clinical attachment loss [56]. This is in accordance with other data concluding that high indices of periodontitis were associated with autoimmune disease [53].

Periodontitis occurs primarily in diffuse SSc [57]. In SSc, periodontitis was characterized by low bleeding on probing values, normal probing depths, and a higher prevalence of gingival recessions [57].

The periodontal ligament space widening on panoramic radiographs is considered by some authors a measure of periodontal damage in SSc [60,61]. Generalized periodontal ligament space widening, as assessed by cone beam computer tomography, is reported in up to half of the patients with SSc [58]. More comprehensive analyses [62] suggest that periodontal ligament widening is the second most frequent oral manifestation in patients with SSc.

Several studies reported a higher prevalence of periodontitis in SSc [20,63], defined according to a well known case definition system [9,64].

A very recent meta-analysis of case-control studies assessing oral manifestations in SSc included 11 studies evaluating periodontal damage and demonstrated a higher prevalence of periodontitis in SSc (Odds Ratio 7.007; 95%CI [3.529, 13.915]), a higher periodontal parameters such as probing depth (Standard Mean Difference SMD 3.101; 95%CI [1.374, 4.829]), and clinical attachment loss (SMD 2.584; 95%CI [0.321, 4.846]) [2]. No statistical significance was found regarding local inflammation quantified as gingival bleeding index (SMD, 1.054; 95%CI [−0.973, 3.081]). This meta-analysis is the first more exhaustive analysis that quantitively evaluated the association between SSc and periodontitis and periodontitis-related signs. Although this meta-analysis [2] included high-quality studies, their number was small, and their case-control design prevented elucidating the cause-effect relationship between SSc and periodontitis [2].

A report not included in the above-mentioned systematic review highlighted lower serum vitamin D levels in patients with SSc and periodontitis (defined based on 2018 Control of Disease Control/American Academy of Periodontology case definition) compared with controls [65]. The number of teeth and the levels of periodontal indices were significantly dependent on serum vitamin D in SSc patients [65]. However, this study did not directly evaluate the associative relationships between SSc and periodontitis. Vitamin D exerts complex effects, including immunomodulation, maintenance of vascular homeostasis, and antioxidative, anticoagulant and antimicrobial effects that impact both SSc and periodontitis [65,66,67,68]. Several studies demonstrate a protective role of vitamin D on oral tissues, especially during different diseases, such as periodontitis [69].

In some observational studies, the relationships between SSc and periodontitis was analyzed based on some debilitating signs as the expression of SSc. An association between periodontitis and reduced mouth opening was presumed [36], but not confirmed by other reports [70]. Other authors showed that gingivitis is associated with diffuse SSc and impaired manual dexterity [48].

The epidemiological data provided by case-control studies should be interpreted with caution, given their small number and the lack of homogeneity concerning patient characteristics and periodontal parameters. Moreover, the variability in the periodontitis case definition influences the reported prevalence of periodontitis in the SSc cohorts, and the appreciation of epidemiological associative links between periodontitis and SSc. These associative relationships should be viewed from the perspective of all pathophysiologic sequelae that accompany both diseases and are likely to contribute toward and against their evolution; they should not be evaluated in the context of a causal effect [71]. Both SSc and periodontitis are complex diseases resulting from interactions of a complex make-up of factors, external and host-derived, detrimental and protective, each accounting for different fractions in the expression of the disease.

Further well-designed studies are needed to explore the relationship between SSc and periodontitis.

## 5. Diagnosis and Evolution of Periodontitis in SSc

The current classification of periodontal and peri-implant diseases and conditions considers that SSc belongs to the group of systemic disorders that can result in loss of periodontal tissue independent of periodontitis; periodontitis developing in SSc was not discussed as a manifestation of systemic diseases [72].

However, periodontitis has been largely described in SSc as mentioned in the previous sections. As for other periodontitis phenotypes belonging to “PERIODONTITIS” category of diseases, a “periodontitis case” in a SSc patient is based on the diagnostic criteria provided by 2018 classification system [73]: (1) the presence of the interdental clinical attachment loss at ≥2 non-adjacent teeth, or of the buccal/oral interdental clinical attachment loss ≥ 3 mm with pocketing ≥ 3 mm at ≥2 non-adjacent teeth, (2) the clinical attachment loss cannot be ascribed to non-periodontitis causes.

A spectrum of attachment level changes following different destruction patterns have been described in periodontitis, such as: a slow, continuous tissue loss, bursts of relatively rapid periodontal destruction in certain teeth (random burst pattern), and frequent bursts of periodontal destruction during certain periods (multiple burst pattern). Presently, no differentiation between periodontitis forms based upon progression rate of destruction is possible [74]. Recent data indicated a rate of progression in a general population of 0.1 mm per year, with increased values in developing countries than in developed ones, and with a little effect of age or gender on attachment level changes [74]. To our knowledge, there is no specific data reporting the activity and progression of periodontitis in SSc patients. However, an increased progression rate in this category of patients should be expected due to the combined effect of elevated pro-inflammatory cytokines, micro-vascular modifications, suboptimal oral hygiene, and medication [54].

## 6. A Brief Overview of the Pathogenesis of Periodontitis

In periodontitis, the microbial subgingival biofilm induces host-mediated inflammation with subsequent tissue damage, periodontal attachment, and bone loss [75].

**Figure 1:**
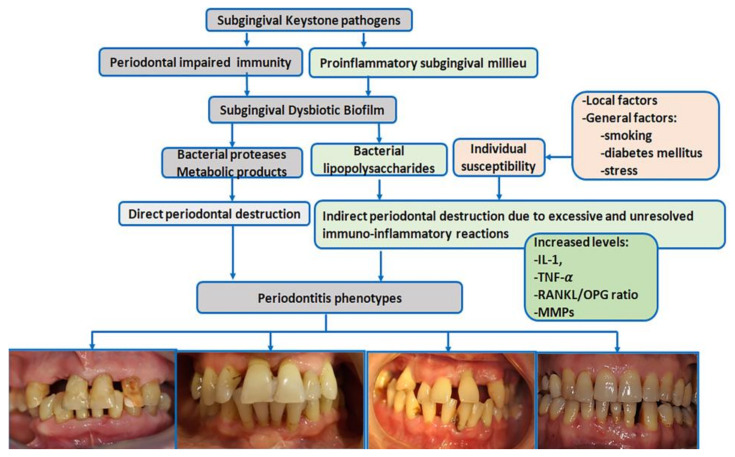
Schematic view of periodontitis pathogenesis (IL-1 = interleukin-1, TNF-α = tumor necrosis factor α, RANKL = receptor activator of nuclear factor kappa-Β ligand, OPG = osteoprotegerin, MMPs = metalloproteinases) [pictures belong to one of authors (AR) periodontitis database].

Periodontitis is a typical biofilm-associated infection [75] in which periodontal biological reactions are modulated by a complex make-up of general factors [Figure 1]. More than 700 different species with distinct subspecies are present in the oral microbiome; many colonize the subgingival areas [76] and deliver essential benefits to general health [76]. Some of these bacteria have been identified as drivers in the pathogenic processes of periodontitis [75].

The ‘red complex’ model is one of the periodontitis pathogenic theories that supports the concept that periodontitis is a multimicrobial disease and acknowledges *Porphyromonas gingivalis*, *Tannerella forsythia*, and *Treponema denticola* as major subgingival pathogens responsible for the development and progression of periodontitis [77]. However, less virulent bacteria have also been linked to periodontitis.

The current polymicrobial synergy and dysbiosis models of periodontitis [75] indicate that the pathogenic driver is a synergistic polymicrobial community in which different members fulfill distinct roles in generating subgingival microbial dysbiosis and host immune disruption [78]. The accumulation of subgingival biofilm induces an inflammatory response where the increased gingival flow and subgingival accumulation of host molecules act as substrates for proteolytic bacteria. These changes in the oral environment cause alterations in microbial proportions and dysbiosis [76], enhancing the risk for periodontitis [78] [Figure 1]. ‘Keystone pathogens’ such as *Porphyromonas gingivalis* modulate the immune response to impair host immune surveillance and tip the balance from homeostasis to dysbiosis [79].

The dysbiotic biofilm releases large amounts of bacterial products, including lipopolysaccharides, which induce the recruitment of immune cells, osteoclast activation, the synthesis of pro-inflammatory cytokines and chemokines, and eventually the destruction of bone and soft tissues [80]. The interleukin-1 (IL-1) and IL-6 family members and tumor necrosis factor (TNF) are pro-inflammatory cytokines with key roles in the progression of periodontitis [81]. IL-1 family members bind to their corresponding receptors, mediate lymphocyte activation, and enhance pro-inflammatory and metalloproteinase (MMP) production destroying periodontal components [81].

The binding between TNF family members and their specific receptors induces a large variety of cell fates, such as death (apoptosis) or life (secretion of pro-inflammatory and osteoclastogenic factors); both drive the destruction of periodontal tissues [80]. TNF-α is locally synthesized by neutrophils, macrophages, and Th1 lymphocytes. TNF-α induces an independent action on receptor activator of nuclear factor kappa-Β ligand, (RANKL) [82] and inhibits osteoblast differentiation and bone nodule formation. In periodontitis, inflammatory cells provide the most abundant expression of RANKL in response to bacterial stimulation [83]. A downregulated expression of osteoprotegerin (OPG)—the soluble decoy receptor that blocks RANKL—has been reported in periodontitis, which increases the RANKL/OPG ratio and, thus, osteoclastogenesis [82].

During periodontal inflammation, the increased oxygen consumption and consecutive local hypoxia induce up-regulation of the transcription of vascular endothelial growth factor (VEGF) within the human periodontal ligament and gingival fibroblast cells. VEGF-A and -C contribute to angiogenesis and lymphangiogenesis in periodontitis [84], but may also accentuate fibrotic changes in SSc.

The mechanisms of periodontitis also include the dysregulation of the resolution phase of periodontal inflammation [85].

## 7. The Biological Pathogenic Links in Periodontitis and SSc

Periodontitis and SSc share common features: both diseases have a comparable disease course, characterized by prominent inflammation during early stages and tissue destruction during advanced stages [54,86].

The etiology of periodontal disease in SSc is most likely multi-factorial and remains elusive [63].

### 7.1. Increased Plaque Formation in SSc Patients

The development of periodontitis in SSc is linked to disease-related factors. Xerostomia promotes dental plaque formation, reduced manual dexterity, and mouth opening as consequences of SSc impair oral hygiene [48]. The accumulation of excessive subgingival biofilm triggers the onset of periodontitis or induces the accelerated progression of already established periodontitis in SSc [54].

### 7.2. The Role of Vasculopathy in Periodontitis Development

Fibrosis and microvascular alterations in SSc are responsible for tissue ischemia and local immune impairments [48]. The SSc vasculopathy also involves the periodontal tissue and may promote periodontitis. Capillaroscopy demonstrates microvascular abnormalities in gingival tissues with a reduced number of capillaries displaying increased diameters and tortuous aspects [87].

VEGF levels are elevated in SSc patients, which may exacerbate fibrotic responses through collagen synthesis and impaired angiogenesis [88]. Excessive serum levels of the anti-angiogenic VEGF165b isoform in these patients could explain the hampered angiogenesis despite elevated VEGF levels [89]. Although histological observations have shown a more intense inflammatory infiltrate in gingival biopsies from SSc patients compared to controls, the expression of VEGF was significantly lower [90]. Controversially, other studies reported an increased expression of VEGF in the gingival tissue and periodontal samples from SSc patients, suggesting that this chronic overexpression could impair the formation of new vessels [91]. Further, the reduced periodontal vascular supply impairs the inflammatory response against periodontal pathogens and decreases local healing capacities.

### 7.3. Proinflammatory Milieu in Both SSc and Periodontitis

High levels of pro-inflammatory cytokines such as TNFα, IL-6, IL-1, and IL-17 are present in the milieu of both periodontitis and SSc [41]. TNFα levels are higher in the gingival crevicular fluid of SSc patients than in controls [53,54]. IL-4, a Th-2 cytokine synthesized by activated TCD8+ lymphocytes and which exhibits a profibrotic effect, was shown to be increased in SSc patient sera [92]. In SSc fibrosis, the chronic inflammatory milieu contributes directly to the myofibroblast differentiation of resident fibroblasts and pre-adipocytes through epigenetic alterations consecutive to DNA methylation and histone modifications [93]. On the other hand, the inflammatory milieu in SSc could exacerbate periodontitis lesions.

Among toll-like receptors (TLRs), TLR4 is a key driver of fibrosis in SSc through the activation of myofibroblasts [94]. The TLR4 ligands in SSc are tenascin-C and fibronectin-EDA, which stimulate collagen gene expression and myofibroblast transformation via TLR4 signaling [94]. High expression of TLR9 in the skin of SSc patients mediates fibrosis through the action of endogenous transforming growth factor-β (TGF-β) [95].

The level of soluble RANKL is increased and associated with osteoporosis in SSc patients [96,97]. RANKL is also an osteoclast-promoting mediator in periodontitis leading to periodontal bone resorption and consecutive tooth loss [98].

### 7.4. Autoimmunity

Antibodies to periodontal collagen and neutrophilic cytoplasm (ANCA) have been reported in periodontitis. SSc patients almost unexceptionally have positive antinuclear antibodies (anti-centromere, anti-topoisomerase, anti-RNA polymerase III, etc.). Positive ANCA were demonstrated in patients with SSc/vasculitis overlap [54].

The dysregulated citrullination phenomenon occurs in both periodontitis and SSc. Peptidylarginine citrullination is an irreversible post-translational protein modification and consists in the conversion of peptidylarginine to peptidylcitrulline. Protein citrullination is catalyzed by the peptidylarginine deiminase (PAD) enzyme family. Peptidylarginine citrullination is considered an important regulator of many physiological pathways, including skin keratinization, myelin formation, gene expression, and the formation of neutrophil extracellular traps, representing the immune system’s first line of defense against infections [99]. The human immune system, in normal conditions, does not self-react to citrullinated proteins. However, as citrullination increasingly affects protein structure and folding, some modified or newly formed epitopes may trigger an immune response against citrullinated hosts [100]. Anti-citrullinated protein antibodies are found in 10% of SSc patients without arthritis and are even more frequently encountered in SSc-rheumatoid arthritis overlap syndrome [101].

Pathological consequences of citrullination in the development of periodontitis have been described [102]. *Porphyromonas gingivalis* peptidylarginine deiminase enzyme (PPAD) can citrullinate both bacterial and human host proteins, inducing an autoimmune response similar to that observed in rheumatic arthritis [102]. Bacterial peptidylarginine deiminase enzyme can spread into the host’s connective tissue and citrullinate epidermal growth factor (EGF), thus blocking its recognition by the epithelium. This pathway delays the local healing process and breaks the local protective epithelial cell-periodontal tissue barrier [103], generating new potent antigenic epitopes. Excessive new epitopes break the tolerance barrier resulting in the generation of anti-citrullinated proteins antibodies (ACPA) [104]. A recent systematic review and meta-analysis [104] reported the presence of ACPA in 9.2% of SSc patients. Although ACPA are less frequent in SSc than in rheumatic arthritis [105], the presence of these antibodies could indicate a pathogenic link with periodontitis. Although autoimmunity is not the major pathogenic pathway in the development of periodontitis, part of periodontal destruction in periodontitis could be attributed to severe autoimmune responses consecutive to citrullinated proteins [102].

The M2 macrophage phenotype contributes to the pathogenesis of SSc by releasing profibrotic cytokines such as TGF-β [94] and vimentin into the extracellular spaces upon activation. The levels of the citrullinated and MMP-degraded metabolite of vimentin (VICM) may be a marker of macrophage activation, tissue degradation, and disease progression in SSc [106].

### 7.5. Vitamin D Involvement in SSc and Periodontitis

Vitamin D is key to the endothelium and vessel homeostasis to maintain vascular health in chronic conditions, including SSc [66]. Vitamin D exhibits antioxidative and anticoagulant effects [67]. Some data reported no differences in vitamin D levels in SSc patients as compared to controls [107]. It is theorized that vitamin D deficiency may accelerate vasculopathy in SSc by stimulating oxidative stress pathways [67]. Moreover, vitamin D modulates the regulation of TGF-β, a crucial mediator in the production of fibroblast and collagen during both SSc and periodontitis [65,68]. Consequently, vitamin D deficiency could interfere with and contribute to the fibrosis of the skin in SSc, which may also cause temporomandibular joints and oral dysfunction [108]. Importantly, SSc patients present several risk factors for vitamin D deficiency, such as skin fibrosis, intestinal malabsorption, insufficient diet, or reduced sunlight exposure [108].

In periodontitis, vitamin D exerts direct, dose-dependent, strong anti-inflammatory action [109], and an antimicrobial effect, especially against the main periodontal pathogenic bacteria [110].

There is data considering that vitamin D contributes to the selective inhibition of *Porphyromonas gingivalis* through the specific gene suppression of the associated virulence factor; this results in the reduction of the *Porphyromonas gingivalis* bacterial load in the gingival biofilm, mitigating the inflammation and damage of the periodontal tissues [110].

## 8. Periodontal Therapy in SSc

**Figure 2:**
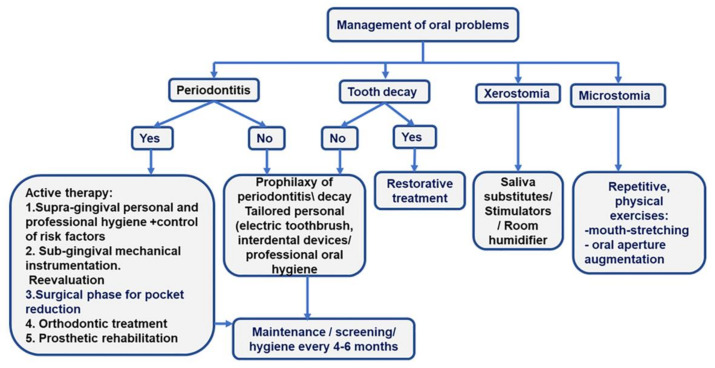
More frequent oral problems in SSc patients and the treatment algorithms.

Three main issues should be considered for the care of SSc patients: oral mucosa involvement, the limitations in mouth opening, and the treatment of related diseases [28]. Prevention of tooth caries and periodontitis is a major objective of oral care in SSc patients. The screening of periodontitis and its detailed diagnosis in the overall picture of oral pathology ensure the development of a comprehensive treatment plan [Figure 2].

- *Preventive therapy.* The management of hyposialia should be carried out by prescribing saliva substitutes and stimulators (pilocarpine hydrochloride if Sjogren’s syndrome is associated) to diminish the risk of periodontitis, oral ulcers, and caries. Daily baking soda mouthwash should also be prescribed.
- *Prevention and management of microstomia.* In patients with microstomia, mouth-opening exercises can improve the inter-incisor distance with severe limitations and the ability to chew, phonation, and dental hygiene [28]. Two times per day, five repetitions of each orofacial physical exercise, holding each for 10 s, should be included in the rehabilitation program of SSc patients: stretching lips with fingers, inflating checks, maximal mouth opening, biting a wood stick with left and right molars, pushing chins to left and right sides with the hand [42].
- *Personal oral hygiene or “patient package”.* The “patient package” can be used to implement a tailored-oral hygiene protocol for efficiently removing tooth biofilm. The “patient package” represents a complex repetitive approach that implies information, motivation, and teaching the use of personalized oral devices (toothbrush and interdental cleaning aids -dental floss and interdental brushes). The oral-cleaning devices are prescribed to accommodate microstomia and decreased manual dexterity. A small-head oscillating-rotating electric toothbrush or a manual toothbrush with a child-sized brush head, used twice daily, are suitable options for SSc patients [48].

Adapted handle-flossing devices or individualized interdental brushes should be used once daily.

- *Management of risk factors*. Cessation of smoking is mandatory.
- *Prevention of tooth caries.* A complex prophylactic package must be implemented to interfere with the cariogenic risk. The package consists of alimentary advice addressing pH modifications, proper specialist-supervised dental hygiene for combating microbial aggression, and systematic fluoridation through fluoride-containing toothpaste associated with fluoride-carrying devices or professional applications of fluoride varnishes [28].
- *Bi-annual dental visits* for screening of oral lesions (complete periodontal, dental, and oral mucosal examination) and maintenance by providing professional hygienic care and, eventually, treatments.

*Specific oral treatments*

- Tooth caries should be treated according to current clinical guidelines.
- If diagnosed, periodontitis treatment should follow current clinical guides [31,32]. Briefly, the first phase of therapy is supragingival hygiene and the control of risk factors; the second phase involves subgingival instrumentation and reevaluation. Surgical approaches, orthodontic treatment, and prosthetic rehabilitation drive patients to the end of active therapy and inclusion in the maintenance phase [Figure 2].
- The use of dental implants is case-dependent, according to the ability to perform oral hygiene, the degree of microstomia, and the insertion of posterior implants [28]. Ideally, anterior implants are recommended, particularly at the canine levels of the jaw, to stabilize a removable metal prosthesis [28]. The implant treatment plan should be tailored with regard to disease severity, the presence of sicca syndrome, limitations in performing oral hygiene, and/or the treatment of an oral cancer. Marked gingival fibrosis and severe microvascular impairment contraindicate dental implants. However, the risk-benefit ratio of implant rehabilitation should be appraised.
- Oral ulcers should be combated by local applications of topical antiseptics and anesthetics (such as chlorhexidine and lidocaine 2%) between meals to limit the risk of aspiration.
- Soft resins may be used to manufacture the base of the removable prostheses to overcome oral lesions induced by rigid materials and microstomia [28].
- Anaesthetics without vasoconstrictors should be used in these patients.

## 9. Conclusions

Patients with SSc have a higher prevalence of periodontitis. SSc and periodontitis may be considered a two-way relationship in which intricated pathogenic mechanisms amplify their evolution. The current data support the hypothesis that SSc and periodontitis act as biologically plausible risk factors for each other.

Maintaining optimal hygiene is an essential target for the prophylaxis of periodontitis and tooth decay, and their treatment.

Daily use of interdental cleaning devices is vital to maintaining good oral status. Identifying proper interdental cleaning devices to surpass hand motricity impairments may increase flossing adherence. Small-head toothbrushes used twice a day are recommended in SSc patients with microstomia.

Depression should be promptly addressed as it may reduce compliance with effective oral hygiene.

## 10. Future Directions

Well-designed clinical case-control studies employing accepted periodontitis case definitions should be designed to evaluate the prevalence and impact of periodontitis in SSc patients, and to define the associative relationships between SSc and periodontitis. Educative programs targeting health care professionals and patients should be developed to prevent and treat periodontitis.

## Data Availability

Not applicable.
